# Investigation of the Crystallization Kinetics and Melting Behaviour of Polymer Blend Nanocomposites Based on Poly(L-Lactic Acid), Nylon 11 and TMDCs WS_2_

**DOI:** 10.3390/polym14132692

**Published:** 2022-06-30

**Authors:** Mohammed Naffakh, Peter S. Shuttleworth

**Affiliations:** 1 Escuela Técnica Superior de Ingenieros Industriales, Universidad Politécnica de Madrid (ETSII-UPM), José Gutiérrez Abascal 2, 28006 Madrid, Spain; 2 Instituto de Ciencia y Tecnología de Polímeros (ICTP-CSIC), Juan de la Cierva 3, 28006 Madrid, Spain; peter@ictp.csic.es

**Keywords:** TMDCs-WS_2_, PLLA, Nylon 11, nanomaterials, morphology, crystallization, melting

## Abstract

The aim of this work was to study the crystallization kinetics and melting behaviour of polymer blend nanocomposites based on poly (L-lactic acid) (PLLA), nylon 11 and tungsten disulfide nanotubes (INT-WS_2_), which are layered transition metal dichalcogenides (TMDCs), using non-isothermal differential scanning calorimetry (DSC). Blends containing different nylon 11 contents ranging from 20 to 80 wt.% with or without INT-WS_2_ were prepared by melt mixing. Evaluation of their morphology with high-resolution SEM imaging proved that the incorporation of inorganic nanotubes into the immiscible PLLA/nylon 11 mixtures led to an improvement in the dispersibility of the nylon 11 phase, a reduction in its average domain size and, consequently, an increase in its interfacial area. The crystallization temperatures of these PLLA/nylon 11-INT blends were influenced by the cooling rate and composition. In particular, the DSC results appear to demonstrate that the 1D-TMDCs WS_2_ within the PLLA/nylon 11-INT blend nanocomposites initiated nucleation in both polymeric components, with the effect being more pronounced for PLLA. Moreover, the nucleation activity and activation energy were calculated to support these findings. The nucleation effect of INT-WS_2_, which influences the melting behaviour of PLLA, is highly important, particularly when evaluating polymer crystallinity. This study opens up new perspectives for the development of advanced PLA-based nanomaterials that show great potential for ecological and biomedical applications.

## 1. Introduction

As research areas, biopolymer science and technology have developed significantly over the last two decades in order to try and address the increasing environmental concerns related to the use of traditional petroleum-based polymers [1,2,3]. Among the various types of polymer matrices used in the processing of structural biocomposites, thermosetting types are the most commonly employed. In this category, the bioderived and biodegradable aliphatic polyester poly (lactic acid) (PLA) is one of the most widely developed and adopted [2]. These materials have in common good biodegradability, renewability and reasonably good mechanical properties and they are easily processed using a standard methodology. The good biocompatibility and bioresorbablity of PLA permit it to be applied for many interesting applications within the pharmaceutical and medical fields, such as in tissue engineering, pharmaceuticals, injury management and drug delivery systems [4]. In addition, the high strength and melting temperature of PLA enable it to be applied as an engineering plastic [2]. By varying the molecular weight and stereochemical composition (L-L, D-D, meso), PLA can possess a Young’s modulus as high as 3 GPa and tensile strength in the range of 50–70 MPa. However, in spite of its many desirable properties, PLA has poor toughness and crystallizability and a low heat distortion temperature, highlighting some of the main challenges that limit its commercial viability [2,3,5,6,7].

In order to rectify some of these deficiencies and extend the application range of PLA, it can be modified via chemical copolymerization, polymer blending or nanocomposite technology. Chemical copolymerization is a very effective way of modifying the properties of homopolymers, and a variety of commercially important copolymers have been achieved via a macromolecular design, as well as chemical copolymerization. In contrast to chemical copolymerization, selection of the appropriate blends produced through physical blending process represents an economic and convenient way of modifying the properties of homopolymers. In the case of PLA, it has been mixed with a wide range of polymers (e.g., polypropylene (PP), poly(methyl methacrylate) (PMMA), poly (butylenes succinate adipate) (PBSA) and nylon 11 [5,6,7]) to generate different properties. Correct tuning of the polymer properties can guarantee that the desired performance of the blends, in terms of toughness, modulus, impact strength, crystallization behaviour, thermal stability, etc., is attained for a particular application. In particular, PLA/nylon 11 blends represent a good option to obtain materials with improved thermomechanical properties compared to neat PLA. Nylon 11 is a bio-based polymer derived from castor oil with excellent thermal stability and high elongation at break and impact strength [7]. However, compatibilization is generally required for these incompatible polymer blends to exhibit the desired properties. The conventional approach to compatibilizing polymer blends is via the use of copolymers, as this is an efficient means to achieve good blend compatibility [8]. However, the lack of commercial availability of specific copolymers and the fact that they must first be synthesized prior to blending is one of their limitations [8]. Incorporation of nanoparticles in order to modify polymers’ interfacial properties and phase morphologies represents another very promising route for the compatibilization of PLA-based blends. In this case, the presence of nanoparticles not only improves the compatibility between the PLA and the blend components but also generates high-performance materials that combine the advantages of the individual polymers within the blend and additionally the benefits of the nanoparticle. Several reviews have been written on the subject and provide a broad overview of PLA-based materials and their properties, demonstrating their many advantages for use within the ecological and biomedical fields [2,3,8,9].

Many promising PLA nanocomposite materials containing synthetic and natural nanoparticles have been developed. For example, carbon nanotubes (CNTs) are a type of anisotropic one-dimensional nanoparticle that have attracted considerable attention in terms of their beneficial effects on the physical properties of PLA. CNTs have also been added along with natural fibres, forming novel PLA composites suitable for various structural applications. In particular, different types of CNTs with specific and unique functional groups have been selected to interact specifically with the hydroxyl groups of cellulose natural fibres and to modify the fibres’ surfaces [10]. With halloysite nanotubes (HNTs), it is possible to design tailored multifunctional materials for use in biomedicine, packaging, corrosion protection and restoration of cultural heritage [11]. For example, Lisuzzo et al. reported coating halloysite nanotubes with chitosan via electrostatic interactions, which proved to be a more feasible strategy to obtain drug delivery systems with tuneable properties [12]. Numerous other potential applications rooted in the compatibility of the nano-bio interface of polymer-HNTs are emerging in the areas of bio-scaffolding, drug delivery and antibacterial treatment [13].

Various methods to enhance the rate of crystallization of PLA have been investigated, as even the fastest crystallizing PLAs are considered slow when compared to many conventional thermoplastics. To this extent, layered transition metal dicalcogenide (TMDC) nanostructures with multidimensional structural anisotropy (0D-IF, 1D-INT and 2D), most notably those of molybdenum and tungsten disulfides (MoS_2_, WS_2_), are fundamentally and technologically intriguing for their versatile properties and applications [14,15,16,17]. The increasing popularity of TMDCs WS_2_ andMoS_2_ over their carbon equivalents is attributed to their low toxicity, biocompatibility [18,19,20,21], relative ease of processing and low cost [22]. In addition, their unique mechanical and tribological properties make them especially appealing as multifunctional platforms for mimicking structural reinforcement for polymer nanocomposites, lubrication, catalysis, rechargeable batteries, solar cells, electronics [14,15,16,17] and, more recently, antiballistic applications [23]. Coupled with these remarkable properties, IF and INT-WS_2_ nanoparticles demonstrate great potential for improving the crystallization rate of PLLA [24,25]. In particular, it was found that the addition of a low concentration of WS_2_ inorganic nanotubes (0.1 wt.%) into PLLA increased its crystallization temperature (T_c_) by up to 17 °C and enabled it to crystallize at a cooling rate as fast as 10 °C/min [24]. However, the incorporation of WS_2_ nanosheets into the biopolymer matrix in fact slows down the rate of PLLA crystallization due to the inactive nucleating role of the 2D-WS_2_ [26]. The explanation for this is that the surface of the WS_2_ nanosheets cannot easily absorb the PLLA chain segments, which in turn greatly hinders crystal growth. Many studies over the years have demonstrated that the size, shape and volume fraction of the additive, as well as other factors, all influence the crystallization processes of confined polymer systems [3]. Understanding the origins of enhanced and retarded crystallization in nanocomposite polymers, including the dynamic mobilities of the different constituents, remains an extremely difficult task [27,28,29].

Hybrid ternary blends comprising two polymers and one inorganic nanofiller are increasingly being studied as a response to various industrial concerns [30]. The object of the current research is to analyse the role of INT-WS_2_ in the morphology, crystallization and melting behaviour of melt-processable PLLA/nylon 11/INT-WS_2_ nanocomposites. In terms of commercial applicability, melt processing is the most economically attractive method for producing polymer blend nanocomposites because it is scalable, versatile and environmentally friendly.

## 2. Experimental Section

### 2.1. Materials and Processing

Poly(L-lactic acid) (PLLA) and nylon 11 were purchased from Goodfellow Ltd. (Huntingdon, UK). Multiwall WS_2_ 1D nanotubes (INT-WS_2_) with diameters of 30–150 nm and lengths of 1–20 μm were obtained from NanoMaterials Ltd. (Yavne, Israel) [24]. All materials were prepared according to the procedure used in our previous work [31]. Briefly, the binary and ternary blend systems of PLLA and nylon 11, with or without INT-WS_2_, were dispersed in a small volume of ethanol (HPLC grade, Sigma-Aldrich Química SL, Madrid, Spain) and homogenized by mechanical stirring and ultrasonication for approximately 15 min. Subsequently, the dispersion was dried in vacuum at 60 °C under a pressure of about 70 mbar for 24 h. The binary (PLLA/nylon 11) and ternary (PLLA/nylon11/INT-WS_2_) blend systems were designated as 80/20, 60/40, 40/60, 20/80, 80/20-INT, 60/40-INT, 40/60-INT and 20/80-INT, where the numbers indicate the weight percentages of PLLA and nylon 11, respectively. The optimum concentration value of INT-WS_2_ used was 0.5 wt.% [24]. The final mixing took place inside a micro-extruder (Thermo-Haake Minilab system) operating at 205 °C with a rotor speed of 100 rpm and mixing time of 10 min. The samples were then pressed into films of 0.5 mm thickness in a hot press system using two heating/cooling plates.

### 2.2. Characterization Studies

The morphology of the cryogenically fractured film surfaces was characterized using an ultra-high field-emission scanning electron microscope (FESEM) (SU8000, Hitachi Co., Tokyo, Japan). All the micrographs were recorded under high vacuum at an accelerating voltage of 3 kV.

Thermogravimetric analysis (TGA) was carried out using a TA Instruments Q50 Thermobalance (Waters Cromatografía, S.A., Cerdanyola del Vallès, Spain) under an inert atmosphere (flow rate = 30 mL/min) at a rate of 10 °C/min up to 600 °C.

Differential scanning calorimetry (DSC) was performed on a Perkin Elmer DSC7/7700 Differential Scanning Calorimeter (Perkin-Elmer España SL, Madrid, Spain), calibrated with indium (T_m_ = 156.6 °C, ΔH_m_ = 28.45 kJ/kg) and zinc (T_m_ = 419.47 °C, ΔH_m_ = 108.37 kJ/kg). Samples with a mass of about 10 mg were encapsulated in standard 50 μL aluminium pans and then placed within a furnace with nitrogen at a flow rate of 25 mL/min, ready to be tested. For the non-isothermal crystallization and melting studies, the samples were first heated to 225 °C and then held at this temperature for 5 min to erase any thermal history. Crystallization of the samples was carried out by cooling, at a rate ranging from 1 to 20 °C/min, from 225 to 40 °C, followed by heating at 10 °C/min over the temperature interval from 40 to 225 °C. The crystallization/melting enthalpy of PLLA in the blend nanocomposites was calculated by considering the weight fraction of PLLA in the nanocomposites. The degree of crystallinity of PLLA and nylon 11 in the PLLA/nylon 11/INT nanocomposites was estimated using the theoretical heat of fusion of 100% crystallized PLLA and nylon 11 (93 J/g [32] and 189 J/g [33], respectively).

## 3. Results

### 3.1. Morphology

The morphology of polymer blend nanocomposites is governed by thermodynamic and/or kinetic effects, as well as the localization of the nanoparticles. For example, when the nanoparticles reside at the interface between two polymers, coalescence can be suppressed and/or interfacial tension reduced, which in turn affects the material’s final properties, such as its mechanical and thermal properties [30,31].

**Figure 1:**
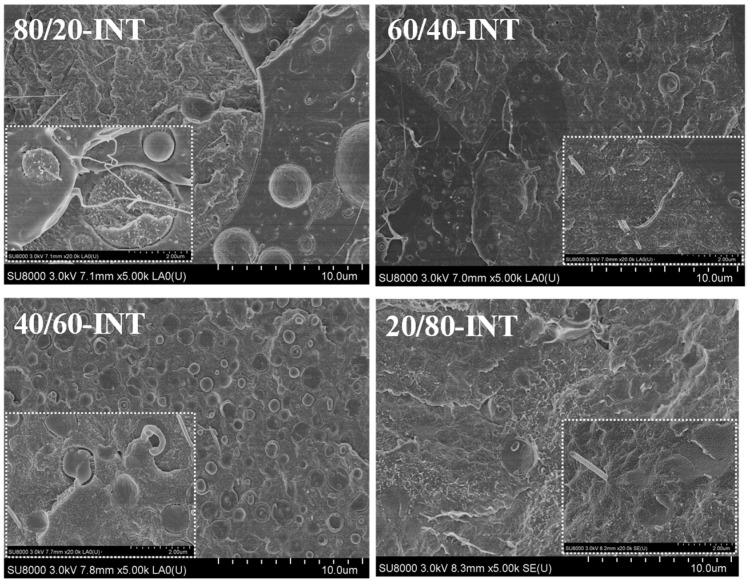
High-resolution SEM micrographs of the PLLA/nylon 11 blends.

Figure 1 shows SEM micrographs of the cryogenically fractured surface morphologies of the melt-processed PLLA/nylon 11 blends. For the 80/20 PLLA/nylon 11 mixture a distinct two-phase morphology was formed, with the minor nylon 11 phase dispersed evenly within the PLLA matrix.

**Figure 2:**
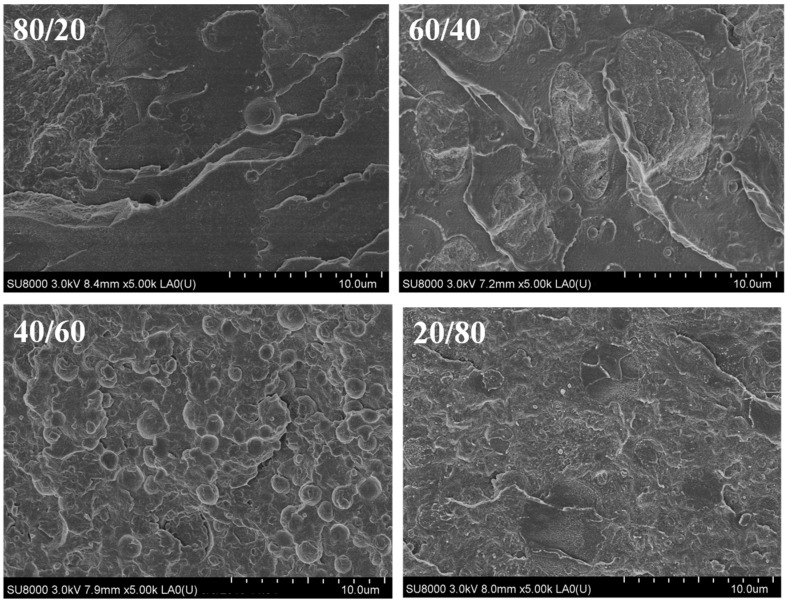
High-resolution SEM micrographs of the PLLA/nylon 11/INT-WS_2_ nanocomposites; the inserts are regions with higher magnification (×20 k), highlighting the WS_2_ inorganic nanotubes and the PLLA–nylon 11 interface.

An increase in the nylon 11 ratio caused the mean diameter of the PLLA/nylon 11 blend domains to also increase. When the PLLA content was ≤60 wt.%, the phase morphology was reversed and the PLLA phase became dispersed within the nylon 11. Furthermore, addition of 0.5 wt.% INT-WS_2_ led to a dramatic reduction in the size of the polymer domains for both the PLLA- and nylon 11-rich blends, as shown in Figure 2, and also improved the compatibility of the two phases. Increasing the magnification to 20,000× revealed the variation in the interface of the PLLA and nylon 11 in the PLLA/nylon 11-INT nanocomposites (e.g., 80/20-INT and 20/80-INT). All these observations suggest that the INT-WS_2_ nanoparticles are uniformly dispersed at the nanoscale without evidence of aggregates or agglomerates, verifying the effectiveness of the melt extrusion process.

### 3.2. Crystallization Behaviour

**Figure 3:**
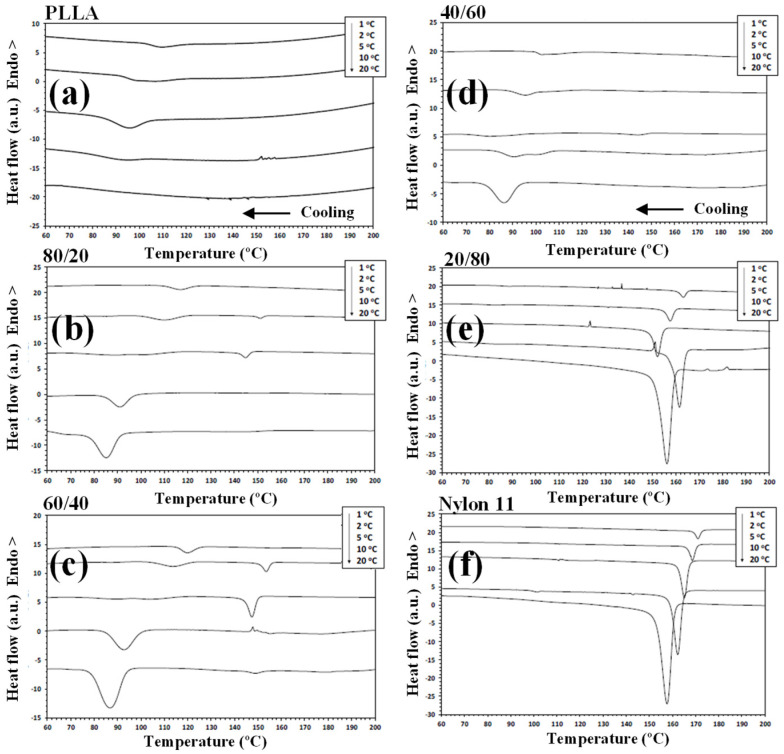
Non-isothermal crystallization DSC thermograms for PLLA, nylon 11 and PLLA/nylon 11 blends at the cooling rates indicated: (**a**) PLLA; (**b**) PLLA/nylon 11 (80/20); (**c**) PLLA/nylon 11 (60/40); (**d**) PLLA/nylon 11 (40/60); (**e**) PLLA/nylon 11 (20/80); (**f**) nylon 11.

**Figure 4:**
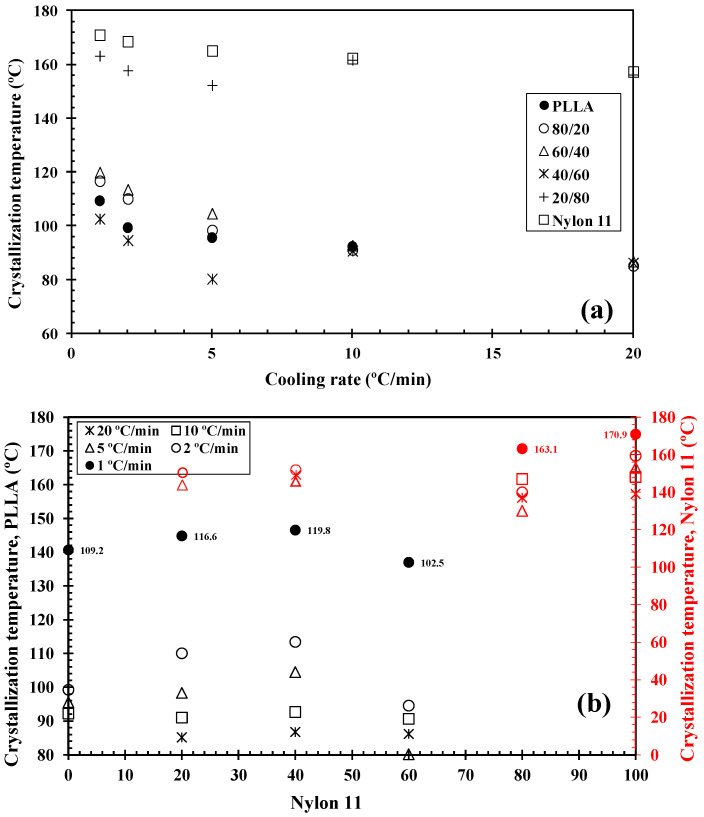
Variation in the PLLA/nylon 11 blends’ crystallization temperatures with (**a**) cooling rate and (**b**) composition.

DSC has been widely used to analyse the crystallization behaviour of PLLA-based materials. It has been found that nylon 11 can crystallize more rapidly and at a higher crystallization temperature than PLLA when it was present within the blend [24,34]. As such, during the cooling segment, nylon 11 separates and crystallizes first. Consequently, any modification to the PLLA/nylon 11 domain interface can potentially influence the phase separation, crystallization rate and final crystal morphology of the polymer blends. Figure 3 shows the DSC thermograms for PLLA, nylon 11, the PLLA/nylon 11 blends and the PLLA/nylon 11-INT blends obtained during non-isothermal crystallization at various cooling rates ranging from 1 to 20 °C/min, corresponding to temperature changes that are found habitually in industrial applications. As can be seen, the crystallization peaks of each of the polymer components became wider and shifted to lower temperatures with increasing cooling rates. This indicates that, at lower cooling rates, the polymer blend components spend longer within the temperature range in order to obtain sufficient mobility in their segments and for crystallization to occur. In particular, it was found that the crystallization peak temperature (*T_p_*) of PLLA was almost undetected at high cooling rates, demonstrating that the crystallization of neat PLLA is very slow. When the ratio of nylon 11 within the blend increased, the PLLA crystallization exotherms shifted to higher temperatures and demonstrated a considerable increase in enthalpy (i.e., the percentage of crystallinity: PLLA = 0%, 80/20 = 14.2% and 60/40 = 31.9% at cooling rate of 20 °C/min). These effects can be more clearly observed in Figure 4, in which the values of the crystallization temperatures of the PLLA/nylon 11 blends are plotted as a function of cooling rate and composition. Pure PLLA and nylon 11 crystallize at approximately 109 °C and 171 °C, respectively, at a cooling rate of 1 °C/min. The crystallization exotherm associated with PLLA could be clearly observed for the blends at the same cooling rate, except for the composition with only 20 wt.% PLLA. In contrast, the apparent exotherm for nylon 11 could be clearly observed at low PLLA concentrations (≤20 wt.%) at cooling rates ranging from 1 to 20 °C/min. The presence of nylon 11 in the blends influenced the crystallization temperature of PLLA, which showed an increase, with the largest difference being observed at a composition of around 40 wt.% nylon 11, indicating that nylon 11 accelerated the melt-crystallization process of PLLA. This nucleating effect on PLLA, resulting from the presence of a second crystallizable polymer, such as PVDF, has been previously reported [31]. The interface between the two phases reduces the surface free energy, facilitating crystal nuclei formation via heterogeneous nucleation. In addition to PDVF crystallization, phase separation can bring about the molecular ordering, alignment, and/or orientation of the PLLA chains at the PLLA/PVDF domain interface via interdiffusion, further aiding crystal embryo development [31].

In contrast, in this case, PLLA caused a decrease in the crystallization temperature of nylon 11 (Figure 4). Both PLLA and nylon 11 are crystallizable and, in this case, PLLA acted as an amorphous fraction (in the molten state), while nylon 11 crystallized. As a consequence of this, crystallization was retarded as PLLA could not act as a solid substrate to help the primary nucleation of the nylon 11. Moreover, the primary nucleation density of nylon 11 was reduced by migration of the nuclei from the nylon 11 phase to the molten phase due to the thermodynamic tendency. These facts played a key role in the variation in the crystallization behaviour of nylon 11 within the PLLA/nylon 11 blends, as the cooling rate varied under non-isothermal conditions for the melt (e.g., 20/80, Figure 3), as well as due to the absence of the apparent exotherms for 40/60 blend systems.

**Figure 5:**
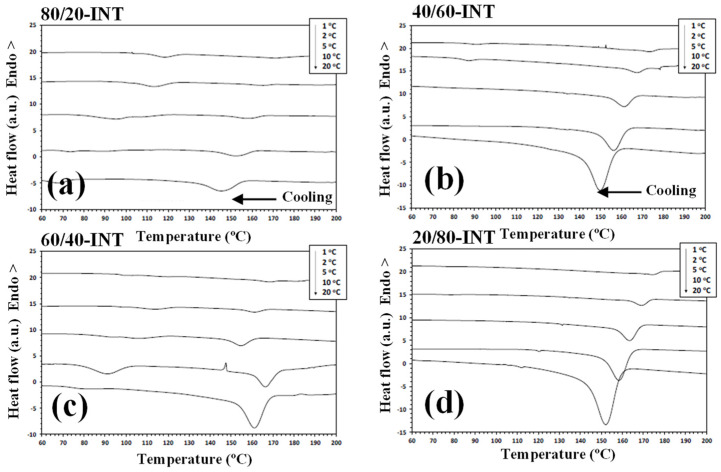
Non-isothermal crystallization DSC thermograms for the PLLA/nylon 11-INT blend nanocomposites at the cooling rates indicated: (**a**) PLLA/nylon 11/INT-WS_2_ (80/20-INT); (**b**) PLLA/nylon 11/INT-WS_2_ (60/40-INT); (**c**) PLLA/nylon 11/INT-WS_2_ (40/60-INT); (**d**) PLLA/nylon 11//INT-WS_2_ (20/80-INT).

**Figure 6:**
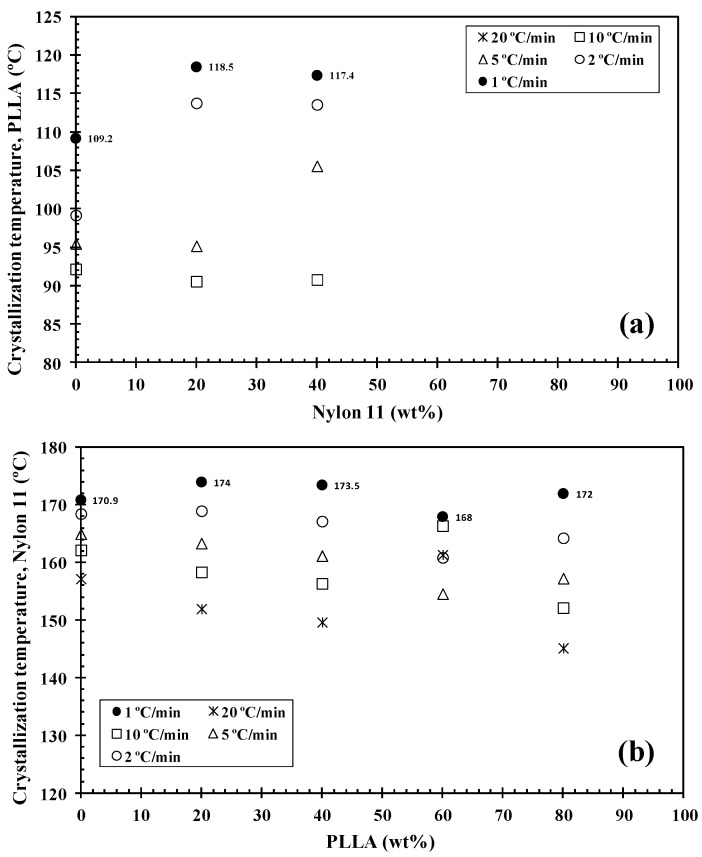
Variation in the PLLA/nylon 11-INT blend nanocomposite crystallization temperatures with (**a**) nylon 11 and (**b**) PLLA concentration.

It is well-known that the crystallization of polymer blend nanocomposites is complex and affected by a variety of factors, including temperature, cooling rate, flow-induced deformation and the presence of a second polymer component, as well as the size, shape and volume fraction of the additive nanoparticles [27,29,30]. Understanding the dynamics of these systems, including the mobilities of the different constituents, remains an extremely a difficult task, despite the wide-ranging research interest in them [29]. Another important feature that should be considered in the study of the crystallization behaviour of these ternary blend systems under dynamic conditions (Figure 5) is that the addition of INT-WS_2_ to PLLA/nylon 11 induced an increase in the crystallization temperature of PLLA and nylon 11, with the increase being higher than 9 °C for the neat PLLA and about 3 °C for the neat nylon 11. To be able to compare this change in the PLLA/nylon 11-INT blends, a graph of *T_p_* vs. the concentration of nylon 11 and PLLA is presented (Figure 6). It can be seen that there was a noticeable development in the crystallization temperature of PLLA when a low cooling rate was employed (i.e., 1 °C/min) and when only 20 wt.% of nylon 11 was added, with the temperature increasing from 109.2 °C to 118.5 °C. It can also be observed that, when the concentration of nylon 11 was further increased, no significant change in *T_p_* could be observed and, obviously, the crystallization temperature was lowered with increased cooling rates. This is also observed for the nylon 11, although it seemed to be less dependent on the concentration of PLLA. The presence of INT-WS_2_ caused an increase in the crystallization temperature of the nylon 11 in the PLLA/nylon 11 blend nanocomposites, with the increase being about 3 °C (*T_p, ylon_*_11_ = 170.9 °C and *T_p,_*_20/80-INT_ = 174 °C). However, the best enhancement was found for the binary PLLA-INT, where the *T_p_* of PLLA increases by more than 20 °C [24]. This suggests that the interface between the two phases reduced the surface free energy. Therefore, the nucleation effect of the INT-WS2 was more dominant in the PLLA/INT nanocomposites than in the PLLA/nylon 11-INT nanocomposites. This discrepancy was likely related to several factors, including the formation of the polymer–polymer interface and its surface energy, roughness and crystalline structure, as well as the filler’s ability to form the critical nuclei [24,31]. All these results appear to demonstrate that WS_2_ inorganic nanotubes can initiate nucleation in both polymeric components within the PLLA/nylon 11-INT blend nanocomposites, with the effect being more pronounced for PLLA.

### 3.3. Nucleation Activity

**Figure 7:**
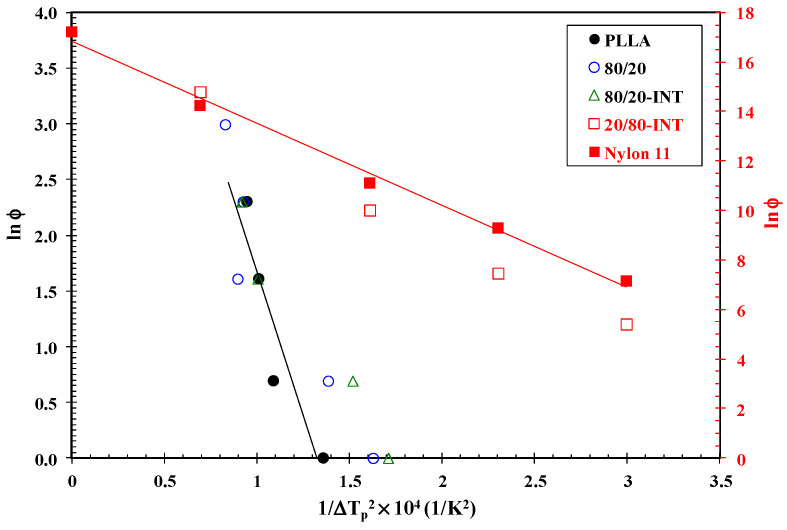
Dobreva plots of PLLA, nylon 11, PLLA/nylon 11 blends and the PLLA/nylon 11-INT nanocomposites obtained from non-isothermal crystallization traces after fitting the data at the cooling rates of 1, 2, 5, 10 and 20 °C/min.

Dobreba and Gutzow [35,36] introduced a semi-empirical model for the determination of the nucleation activity (*φ*) of foreign substrates (such as INT-WS_2_ or another polymer) in polymer melts using DSC measurements. Nucleation activity is a factor describing how the work of 3D nucleation decreases with the presence of foreign particles, which are assigned values varying from 0 to 1, corresponding to extremely active and inert foreign substrates, respectively. Specifically, the more active the nucleator is, the lower the *φ* value. According to this model, *φ* can be calculated as follows:

(1) $$\varphi =\frac{B^{*}}{B}$$

where *B* is a parameter for the pristine polymer and *B*^∗^ is for the polymer/nucleator system. *B* and *B*^∗^ can both be experimentally determined from the slope of the following equation:

(2) $$\ln\varphi =A-\frac{B(or\;B^{*})}{\Delta T_{p}^{2}}$$

where *φ* is the cooling rate, A is a constant, Δ*T_p_* is the amount of supercooling (*T_m_* − *T_p_*), *T_m_* is the melting temperature and *T_p_* is the crystallization peak temperature. It is clear that, as the cooling rate increases, *T_p_* shifts to lower temperatures both for the neat PLLA and the PLLA/nylon 11 blends and nanocomposites. A linear relationship was obtained for each sample, as can be observed in Figure 7, considering a value of 195 °C for the thermodynamic equilibrium melting temperature of PLLA [24] and 202.8 °C for nylon 11 [34]. The values of *B* and *B*^∗^ were obtained from the slope of the fitted lines, and the nucleation activity (*φ*) was calculated from this ratio. Thus, the values of *φ* for the PLLA/nylon 11 blends containing 20 and 40 wt.% nylon 11 were calculated to be 0.63 and 0.61, respectively. More importantly, the addition of INT-WS_2_ reduced the *φ* values (*φ*_80/20-INT_ = 0.40 and *φ*_60/40-INT_ = 0.61) compared to PLLA/nylon 11 blends without the added nanofiller, indicating that the presence of INT-WS_2_ further accelerated the PLLA crystallization process. The previously mentioned nucleation effect that led to the increase in crystallization rate for PLLA is highly important in the case of nylon 11, particularly when evaluating the tendency for nucleation activity in the PLLA/nylon 11-INT nanocomposites. All nanocomposites had a reduced nucleation activity factor (*φ*_20/80-INT_ = 0.59, *φ*_40/60-INT_ = 0.59 and *φ*_20/80-INT_ = 0.62) in comparison to the PLLA/nylon 11 blends (*φ* = 1), which means that they had enhanced 3D nucleation and were active as heterogenous nucleating agents. The trend observed for *φ* was consistent with the trend seen for the variation in crystallization temperature for nylon 11 in the PLLA/nylon 11-INT blends, implying that INT-WS_2_ is an active nucleating agent for the non-isothermal melt crystallization of PLLA/nylon 11.

### 3.4. Crystallization Activation Energy

**Figure 8:**
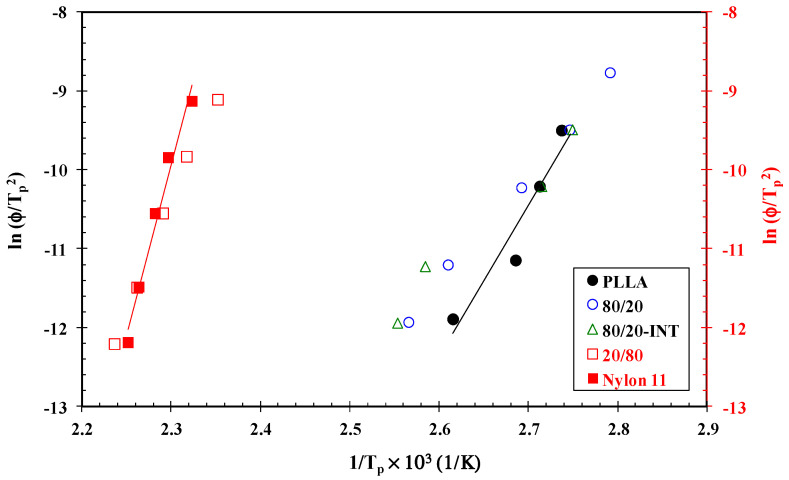
Kissinger plot for PLLA, nylon 11, PLLA/nylon 11 blends and PLLA/nylon 11-INT nanocomposites obtained from non-isothermal crystallization traces after fitting the data at the cooling rates of 1, 2, 5, 10 and 20 °C/min.

The activation energy values obtained from the non-isothermal crystallization thermograms of PLLA and its blends and nanocomposites were calculated using the Kissinger method. This approximation theory determines the activation energy for the transport of the macromolecular segments to the growing surface, which is determined from the maximum conversion rate. The corresponding formula is [37]:

(3) $$\ln\frac{\varphi }{T_{p}^{2}}=\mathrm{Constant}-\frac{\Delta E}{RT_{p}}$$

where *R* is the universal gas constant. The activation energies were calculated using the slopes of the lines obtained from plots of log $\varphi /T_{p}^{2}$ vs. 1/*T_p_* (Figure 8). Thus, the values of Δ*E* for neat PLLA and its blends containing 20, 40 and 60 wt.% of nylon 11 were calculated to be −159.2, −100.9, −107.6 and −116.4 kJ/mol. Moreover, the presence of both nylon 11 and INT-WS_2_ increased the activation energy of the PLLA/nylon 11-INT compared to the PLLA/nylon 11 (Δ*E*_80/20-INT_ = −63.1 kJ/mol and Δ*E*_60/40-INT_ = −102.9 kJ/mol) blends without nanoparticles. According to this, the restriction of the transport of the PLLA macromolecular segments to the growing surface did not appear to be a limiting factor in the crystallization rate, demonstrating that the nucleation activity of the inorganic nanotubes played a dominant role in accelerating the crystallization of PLLA. Likewise, the values of Δ*E* of nylon 11 experienced a large increase from −369.4 kJ/mol to −94.8 kJ/mol with the addition of both PLLA and IN-WS_2_ (Δ*E*_20/80-INT_ = −226.5 kJ/mol, Δ*E*_40/80-INT_ = −211.4 kJ/mol, Δ*E*_60/40-INT_ = −94.8 kJ/mol and Δ*E*_80/20-INT_ = −185.8 kJ/mol). These results agree with the results for the non-isothermal crystallization kinetics of nylon 11 and confirm the nucleating activity of the additive, INT-WS_2_.

### 3.5. Melting Behaviour

**Figure 9:**
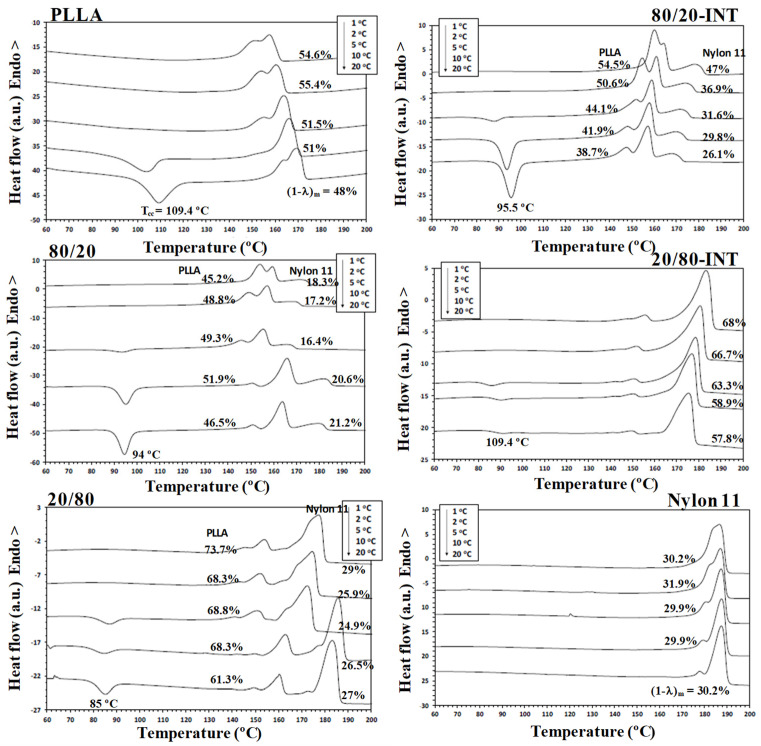
DSC thermograms obtained at a heating rate of 10 °C/min for the PLLA/nylon 11-INT blend nanocomposites after non-isothermal crystallization at the indicated cooling rates.

Melting of biopolymer blend nanocomposites is a very complex process that is heavily influenced by the conditions under which crystallization occurs [24,31]. The DSC heating curves for the samples recorded at 10 °C/min, subsequent to crystallization from the melt at different cooling rates as indicated, are shown in Figure 9. It can be observed that the neat PLLA sample exhibited exothermic peaks attributable to the process of cold crystallization, which was influenced by the cooling rate and the nylon 11 loading. The appearance of these exothermic peaks for the samples crystallized at higher cooling rates indicated that the melt-crystallization process was incomplete. In particular, the cold-crystallization temperature (*T_cc_*) values of PLLA decreased with the addition of nylon 11 due to the nucleating effect of nylon 11 on the crystallization of PLLA (e.g., *T_cc, PLLA_* = 109.4 °C and *T_cc_*_, 80/20_ = 94 °C). Moreover, it is interesting to note that the first endothermic peak area decreased whereas the second endothermic peak area increased with faster rates of cooling. This implies that the peak at higher temperatures mainly arose from the rearrangement of the initial crystal morphology (i.e., melting–recrystallization–melting) and that the peak at lower temperatures represented the melting of the original crystals formed when the sample was cooled from the melt [24]. The aforementioned results are reasonable because the time for PLLA to crystallize became shorter with increasing cooling rates. Thus, the crystals formed during non-isothermal crystallization were not as perfect or stable and, therefore, recrystallized and were reorganized into more perfect, more stable crystals during the subsequent heating scan. As such, when high cooling rates were used, the second endothermic peak grew and became more dominant. The analogous data for the double-melting peaks versus the cooling rate with nylon 11 concentration, using the presence of INT-WS_2_ as a parameter, are shown in Figure 9.

In a similar manner, the cooling rate also influenced the crystallinity of the PLLA systems. Figure 9 shows the evolution of the PLLA/nylon 11/INT-WS_2_ nanocomposite crystallinity (1 − *λ*)*_m_* calculated from the double endothermic curves with different cooling rates and compositions. As can be seen, the increase in cooling rate progressively reduced (1 − *λ*)*_m_*, as the polymer chains had less time to organize into crystalline domains with fewer defects. The previously mentioned nucleation effect of nylon 11 that led to it influencing the melting behaviour of PLLA is highly important, particularly when evaluating the tendency toward melting crystallinity. In particular, the (1 − *λ*)*_m_* values for the binary PLLA/nylon 11 blends were higher than those of neat PLLA. For example, the crystallinity of PLLA changed from 48–55% to 65–75% when 40% nylon 11 was present and continued to increment with higher nylon 11 concentrations. Likewise, the (1 − *λ*)*_m_* of the PLLA/nylon 11-INT nanocomposites showed a similar trend as a function of *φ* and exhibited lower values than the PLLA/nylon 11 blends. These results also confirm that the role of INT-WS_2_ in the variation of the (1 − *λ*)*_m_* values for PLLA appears to be less relevant with the presence of nylon 11. However, in the case of nylon 11, the addition of INT-WS_2_ to the PLLA/nylon 11 blends induced an increase in the crystallinity values of these components, the increase being more pronounced for the nanocomposite blends rich in nylon 11 (20/80-INT).

## 4. Conclusions

In this work, inorganic nanotubes (INT-WS_2_) were incorporated into PLLA/nylon 11 blends via simple melt processing, and the morphology, crystallization and melting behaviour of the resulting nanocomposites were investigated. Additions of nylon 11 and 1D-TMDCs WS_2_ were found to be effective as novel routes to producing advanced PLLA/nylon 11 nanocomposites made via the widely used melt process. From the SEM images, it was observed that the nanofiller was well-dispersed, which helped modify the blend interface morphology. The temperature and crystallization rate of nylon 11were higher and faster, respectively, than those of PLLA. Furthermore, it was found that, when blended with PLLA, there was an important effect on the rate of PLLA crystallization, which increased, and this in turn was influenced by the cooling rate. In contrast, analysis of the crystallization behaviour of the second component, nylon 11, showed that its crystallization temperature decreased in the presence of PLLA. Furthermore, it was found that the incorporated nanofiller INT-WS_2_ had a nucleating effect on both the PLLA and nylon 11, although this effect was more prominent for PLLA and the PLLA-rich blends, reflected not only by an increase in the crystallization temperature but also in crystallinity. More importantly, investigation of the nucleation activity using the Gutzow and Dobreva model revealed that the addition of 1D-TMDCs WS_2_ played a fundamental role in the promotion of PLLA crystallization. In the subsequent heating, the complex endothermic melting peaks for PLLA and nylon 11 were attributed to a melt-recrystallization mechanism. The developed crystallinity of PLLA and nylon 11 was found to be influenced by the cooling rate and composition. These results have considerable practical significance for the technological processing of PLLA/layered transition metal dichalcogenide (TMDC) nanocomposites.
